# Clear cell change in eccrine sweat glands noted in frozen section

**DOI:** 10.1002/hsr2.265

**Published:** 2021-03-09

**Authors:** Nicole Edmonds, Vernon Forrester, Darren Guffey, Mark Russell

**Affiliations:** ^1^ Department of Dermatology University of Virginia School of Medicine Charlottesville Virginia

A 51‐year‐old male underwent Mohs micrographic surgery (MMS) for a 6 mm infiltrative basal cell carcinoma of the right sideburn. Review of fresh frozen sections (FFS) during MMS did not reveal any residual tumor but was notable for clear cell metaplastic change in the adjacent eccrine sweat glands (Figure 1). Following the procedure, additional tissue from the resection was sent for permanent section (PS) analysis in order to further characterize the nature of the clear cell change. On PS, the clear cells were periodic acid‐Schiff positive and diastase labile indicating the presence of glycogen.

**FIGURE 1:**
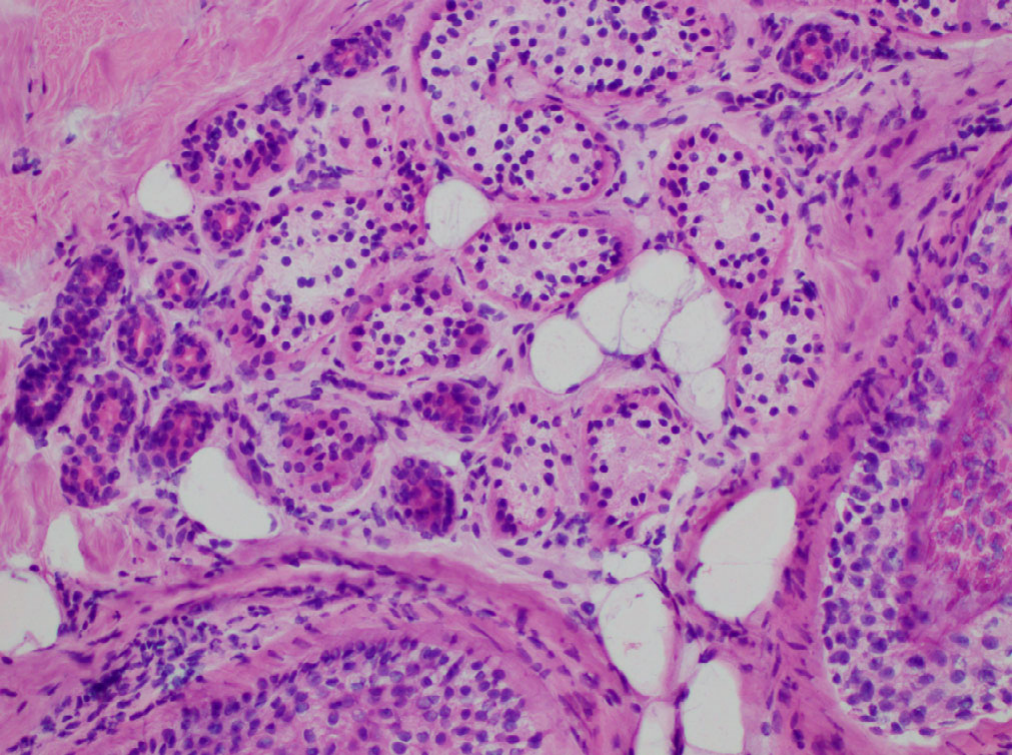
FFS, H&E, 20× magnification. Note the predominance of clear cells with a finely granular cytoplasm within the secretory portion of the eccrine gland

While most eccrine glands have three different cell types in the secretory coil (clear cells, dark cells, and myoepithelial cells), some eccrine glands contain only clear cells, which are referred to as “clear cell change”.^1^ The clear cell changes in this case were isolated to the eccrine glands and were therefore favored to represent a benign variant of normal histology. The high glycogen content of the clear cells had previously been hypothesized to be the result of deficient glycogen phosphorylase activity such as in diabetes,^2^ but Cherian et al disproved this hypothesis.^1^

Clear cell change in eccrine glands has been reported in 0.5% of biopsy specimens analyzed with PS.^1^ To the authors' knowledge, this phenomenon has never been reported on FFS, the technique employed during MMS. This discrepancy in prevalence of clear cell change may be related to differences in tissue processing, with superior cellular detail attainable on PS. Underreporting of this feature in the literature could also account for this inconsistency. Nevertheless, Mohs surgeons should be made aware of this possible incidental finding to prevent additional resection out of concern for tumor burden or submission of additional tissue for pathological analysis.

## FUNDING

There was no supporting source/financial while conducting this study.

## CONFLICT OF INTEREST

The authors have no conflicts of interest to disclose.

## TRANSPARENCY STATEMENT

The lead author (Nicole Edmonds), affirms that this manuscript is an honest, accurate, and transparent account of the study being reported; that no important aspects of the study have been omitted; and that any discrepancies from the study as planned (and, if relevant, registered) have been explained.

## AUTHOR CONTRIBUTIONS

Conceptualization: Nicole Edmonds, Darren Guffey, Vernon Forrester, Mark Russell

Investigation: Nicole Edmonds

Project Administration: Darren Guffey, Vernon Forrester

Supervision: Mark Russell

Writing – Original Draft Preparation: Nicole Edmonds

Writing – Reviewing and Editing: Nicole Edmonds, Darren Guffey, Vernon Forrester, Mark Russell

  The corresponding author had full access to all of the data in the study and takes complete responsibility for the integrity of the data and accuracy of the data analysis.

### ETHICS STATEMENT

A signed informed consent was obtained from the patient and attached to this manuscript.

## Data Availability

The authors confirm that the data supporting the findings of this study are available within the article [and/or] its supplementary materials.
